# Development of patient-centric conceptual frameworks for symptoms and impacts of ornithine transcarbamylase deficiency (OTCD)

**DOI:** 10.1186/s41687-025-00939-5

**Published:** 2025-10-22

**Authors:** Christina Theodore-Oklota, Ashley O’Mara, Jessica Butler, Shayna Egan, Elizabeth Hribal, Christopher Evans

**Affiliations:** 1 Ultragenyx, 60 Leveroni Court, Novato, CA 94949 USA; 2 Lumanity, 280 Summer Street, 8th floor, Boston, MA 02210 USA

**Keywords:** Ornithine transcarbamylase deficiency, Urea cycle disorder, Patient-reported outcomes

## Abstract

**Background:**

Ornithine transcarbamylase deficiency (OTCD) is a rare hereditary metabolic disorder caused by mutations in the *OTC* gene. It is the most common urea cycle disorder (UCD), occurring in approximately one in 14,000 births worldwide. As an X-linked enzyme, OTC breaks down citrulline in mitochondria, and pathogenic variant can lead to dangerously high levels of ammonia (hyperammonemia) and glutamine (hyperglutaminemia) in the blood. Symptoms related to hyperammonemia include confusion, vomiting, coma, and cognitive impairments. Current treatments include ammonia scavengers and strict dietary management to limit protein intake.

**Objectives:**

Given the rarity of OTCD and its varied clinical presentation, this study aimed to understand and characterize the burden of disease from the patient and caregiver perspective, develop preliminary symptoms and impacts conceptual frameworks based on the patient experience and caregiver observations, and contribute to the development of new clinical outcome assessments (COAs). The development of patient-centric conceptual frameworks is important to ensure that the unique experiences and needs of patients are accurately reflected in research and healthcare strategies, ultimately leading to more effective and personalized treatment approaches and disease measures.

**Methods:**

The research involved a comprehensive literature review, interviews with key opinion leaders (KOLs), and concept elicitation interviews with patients and caregivers. These methods were employed to identify and validate relevant symptoms and impacts of OTCD.

**Results:**

The most commonly reported symptom domains (i.e. neurocognitive, gastrointestinal, energy-related [fatigue/tiredness], and physical symptoms) and impact domains (i.e. diet-related, emotional, physical, sleep, and social problems) reported by patients, caregivers, and KOLs were included in the preliminary conceptual frameworks. The research suggests that there is a high humanistic burden on patients that may be difficult to manage and negatively impacts quality of life for those living with OTCD. The findings of this study indicate that these frameworks encompass the concepts most important to patients and are aligned with Food and Drug Administration (FDA) guidance on human gene therapy for rare diseases which ensures that measures are scientifically valid, reliable, sensitive and responsive when used in clinical trials of novel treatments to document treatment benefit.

**Conclusion:**

This research highlights the need for COAs specific to OTCD based on the patient perspective in this disease population. This work helps to define the humanistic burden of disease and lays the foundation for identifying and developing robust COAs tailored to improve clinical assessments and patient care in OTCD.

**Supplementary Information:**

The online version contains supplementary material available at 10.1186/s41687-025-00939-5.

## Background

Ornithine transcarbamylase deficiency (OCTD), the most common urea cycle disorder (UCD), is a rare hereditary metabolic disorder caused by mutations in the *OTC* gene, affecting approximately one in 14,000 births worldwide [1]. Most current research focuses on urea cycle disorders (UCDs) in general rather than on OTCD specifically due to the shared pathophysiology (i.e. functional and biochemical changes associated with or resulting from diseases or disorders) and clinical features, the overlap in therapeutic targets, and the resource efficiency associated with studying UCDs collectively; however, OTCD poses unique challenges due to its genetic variability, and related heterogeneity in clinical presentation, as well as its inheritance pattern [1]. OTC is an X-linked enzyme that breaks down citrulline from carbamoyl phosphate and ornithine in the mitochondria, so mutations can lead to deficient enzyme activity and high ammonia blood levels that prevent the usual flow of ammonia through the urea cycle, resulting in dangerously high levels of ammonia (hyperammonemia) and glutamine (hyperglutaminema) in the blood [1–3]. These disruptions may result in severe symptoms such as confusion, vomiting, coma, cognitive functioning impairments that are cumulative and irreversible, and death [1, 3, 4].

Typically, this disorder manifests more severely in newborn males than females, though late-onset disease may occur in both through adulthood [2]. Symptoms in infants, appearing 4–48 hours after birth, include encephalopathy, difficulty eating, lethargy, ataxia (i.e. lack of coordination), seizures, hypothermia, apnea (i.e. breathing stops temporarily), and respiratory alkalosis [1]. Diagnosis can be confirmed using biochemical and genetic testing, and recent research has identified methods to predict disease severity in males with OTCD [5, 6].

Treatment of OTCD typically involves managing hyperammonemia events, which includes immediate restriction of protein intake and increase in the intake of carbohydrates and lipids, hemodialysis for patients in hyperammonemic coma, and administration of sodium benzoate, arginine, L-arginine, dextrose, intralipids, and sodium phenylacetate [7–9]. Management of OTCD focuses on preventing acute and chronic complications through dietary restrictions (low-protein diet), essential amino acids, vitamin and mineral supplementation, medications to increase waste nitrogen excretion, and, in some cases, liver transplantation [10]. However, current dietary and medical treatments leave high unmet needs in terms of preventing hyperammonemia, and the only curative option, liver transplantation, is not widely available. Therefore, gene therapy has emerged as a new area of promise for treating and potentially curing OTCD using both viral and non-viral vector strategies.[11]

UCDs, including OTCD, have been shown to lead to substantial physical and psychosocial impacts in children, [12] requiring comprehensive mental health care, psychological support, and metabolic management. Although research on the impact on overall health-related quality of life (HRQoL) is mixed – potentially due to the use of generic HRQoL assessments in studies – attention problems and emotional and behavioral issues are often reported [13 Prior research with 66 surveyed clinicians who actively managed at least one patient with UCD, with some managing patients with OTCD specifically, indicated that over 90% of said clinicians would describe UCDs, including OTCD, as “very or extremely burdensome” to patients and their families due to the need for strict adherence to therapies.[14]

Despite its prevalence among UCDs and appealing candidacy for gene therapy, OTCD from the patient perspective remains poorly understood due to its rarity and heterogeneity. This research sought to better understand the patient experience of living with OTCD and develop preliminary conceptual frameworks for symptoms and impacts of OTCD to guide the identification of relevant clinical outcome assessments (COAs) or the development of new COAs. COAs are measures that describe or reflect how a patient feels or functions and can include patient-reported outcome (PRO), observer-reported outcome (ObsRO), clinician-reported outcome (ClinRO), or performance outcome (PerfO) measures or questionnaires. Since generic HRQoL COAs are designed to assess a wide range of populations and conditions, they often fail to adequately capture the unique aspects of OTCD, and potentially overlooking important impacts on quality of life [15]. This study is novel in its approach of specifically focusing on OTCD from the patient and caregiver perspectives and developing OTCD-specific conceptual frameworks, since OTCD has largely been grouped with UCDs as a whole in other research. While the approach to researching UCDs collectively is important for gaining insights to the shared pathophysiology and broader clinical and treatment-related implications, research specific to OTCD is vital to understanding the unique aspects of the disorder and developing more targeted therapies and measurement strategies.

## Methods

### Targeted literature review

Two focused literature reviews were conducted via Medline^®^: the first to identify clinician- and patient-reported signs, symptoms, and impacts of OTCD, and the second to find potentially relevant COAs. Searches were limited to articles published from 2009 to 2019 (and later updated to 2024), English language, and human subjects only. Search terms used in the literature review can be found in Appendix I. Following the searches, MS Excel was used to track the review of each abstract. The articles were evaluated by predetermined inclusion and exclusion criteria. Inclusion criteria included:

- Clinician-report or patient-report of a sign, symptom, or impact of OTCD was included or described in the article; or
- Article focused on instruments developed using clinician or patient input to measure signs, symptoms or impacts of OTCD.

Exclusion criteria included:

- Relevant OTC term included in the title but not the main focus of the article; or
- Article focused on unusual presentations of UCDs (i.e. case studies) or specific populations (e.g. UCDs in pregnant women); or
- Article was a systematic review containing and citing information included in other articles (i.e. repetitive information); or
- Article reported on purely objective, physiological measures of OTCD (e.g. medical imaging).

Data extraction was done systematically to inform the specific research objectives, and articles selected for full review were coded using Atlas.ti version 8.4.20.0. Relevant information such as population details (e.g. patients with OTCD, other UCDs, gender, onset type) was tagged. Patient-experienced concepts were extracted, summarized, and considered for the conceptual framework, with their frequency of mention documented.

The following regulatory documents were also reviewed: Draft Guidance on Inborn Errors of Metabolism that Use Dietary Management (2018) [16] and Workshop on Assessing Neurocognitive Outcomes in Inborn Errors of Metabolism (2015) [17] and were considered when developing the preliminary conceptual frameworks.

### Key opinion leader interviews

Key opinion leaders (KOLs) with experience treating patients with OTCD were identified by the study sponsor and those that expressed interest in participating in an interview to provide input on concepts for inclusion in the preliminary frameworks were introduced to the research team for interview scheduling. KOLs were interviewed via telephone by trained and experienced interviewers using a semi-structured interview guide. Interview topics focused on the presentation (i.e. signs/symptoms and impacts), the treatment options for all UCD disorders with an emphasis on OTCD, and any recommendations for potential concepts that should be included in existing or future COAs. Interviews were audio recorded, transcribed by a transcriptionist, and coded using Atlas.ti version 8.2.34.

### Initial concept elicitation interviews in UCDs

Following ethics approval (Western-Copernicus Group [WCG] institutional review board [IRB] Protocol #20190078), a Recruitment Flyer was distributed by KOLs and an OTCD advocacy organization, and posted via various social media channels, to recruit patients with UCDs for the initial concept elicitation interviews. Interested participants were screened via telephone and asked to provide details pertaining to how they learned about the research study and symptoms associated with hyperammonemia to confirm eligibility.

The main clinical study eligibility criteria for children and adults with UCDs were as follows:

- Caregiver’s child or adult participant had been diagnosed with one of the following UCDs: OTCD, adenylosuccinate lyase deficiency (ASLD), arginine-1 deficiency (ARGD), carbamoyl phosphate synthetase 1 deficiency (CPS1D), citrin deficiency, hyperornithinemia-hyperammonemia-homocitrullinuria (HHH) (or ornithine transporter 1 deficiency [ORNT1D]), or N-acetylglutamate synthetase deficiency (NAGSD);
- Adult participant or caregiver confirmed they or the child with OTCD had ever experienced a hyperammonemia event that included at least one of the following: confusion or abnormal cognition, vomiting, abnormal appetite, ataxia, or seizures **AND/OR** caregiver confirmed their child was currently being treated for their UCD with a restricted diet or ammonia scavenger medication;
- Adult participant or caregiver confirmed they or the child with OTCD had not received a liver transplant.

If an eligible caregiver had a child or adolescent (aged 8–17 years) with a UCD, the child or adolescent was also asked to participate in an interview; the caregiver was asked to confirm if their child was willing and able to participate in a 90-minute interview during which they would be asked to describe their experience regarding signs, symptoms, and impacts of their UCD. All participants were located in the United States.

Eligible participants completed 90-minute interviews via telephone or in-person with a trained interviewer guided by a semi-structured interview guide. Interview guide topics included open-ended questions intended to facilitate spontaneous elicitation of the signs/symptoms, impacts and experiences related to UCDs, including OTCD, from the participants’ perspectives. Participants were compensated upon completion of their interview. Interviews were audio recorded, transcribed by a transcriptionist, and coded using Atlas.ti version 8.4.19.

The decision to initially recruit participants with other UCDs to participate in the concept elicitation interviews was made due to the rarity of OTCD, which contributes to recruitment challenges, as well as the overlap in biochemical and clinical features across UCDs. However, a target was set for the interview sample of at least 50% of participants with OTCD. Later, changes were made to the eligibility criteria to restrict participation to only those with OTCD due to concerns that the data for UCD participants may be highly divergent and analyzing UCDs in aggregate would detract from the primary focus of OTCD-specific experiences.

**Fig. 1:**
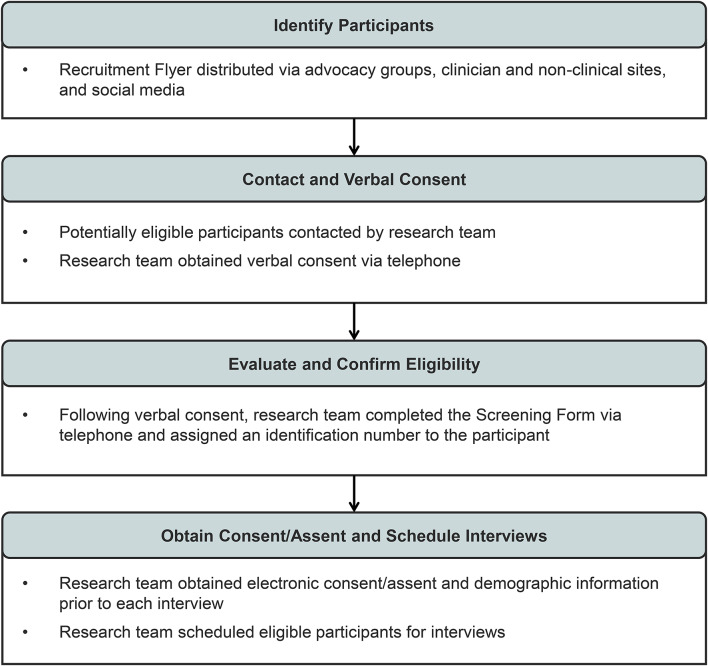
Recruitment process for patients and caregivers

The recruitment process for caregivers and patients with OTCD is shown in Fig. 1.

### Development of the preliminary conceptual frameworks for potential PRO measures

**Fig. 2:**
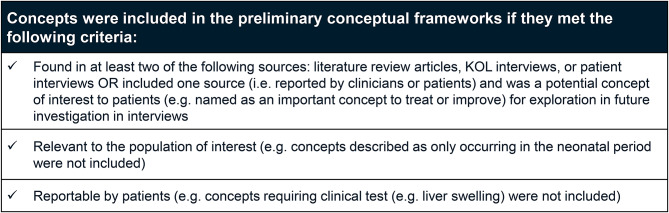
Inclusion criteria for preliminary conceptual frameworks

A conceptual framework is a diagram that illustrates the relationships between items, domains, and concepts measured by an instrument. The initial version is hypothesized and evolves as empiric evidence is collected to support the actual relationships [18]. The development of the preliminary conceptual frameworks was informed by the results of the literature review, KOL interviews, and concept elicitation interviews with patients with OTCD. Inclusion criteria used to determine the concepts in the preliminary conceptual frameworks are shown in Fig. 2.

### Additional concept elicitation interviews in OTCD

Following the development of the initial conceptual frameworks, additional interviews were conducted with patients and caregivers of patients with OTCD to help confirm the concepts included in the preliminary conceptual frameworks. The eligibility criteria were identical to that of the initial concept elicitation interviews with the exception that participation was restricted to those with OTCD only to support the research objectives of capturing patient experiences and caregiver observations specific to OTCD. Interviews were conducted and analyzed in a similar manner to the first round and the conceptual frameworks were revised based on participant feedback. Interview probes are included in Appendix II.

## Results

### Literature review

**Fig. 3:**
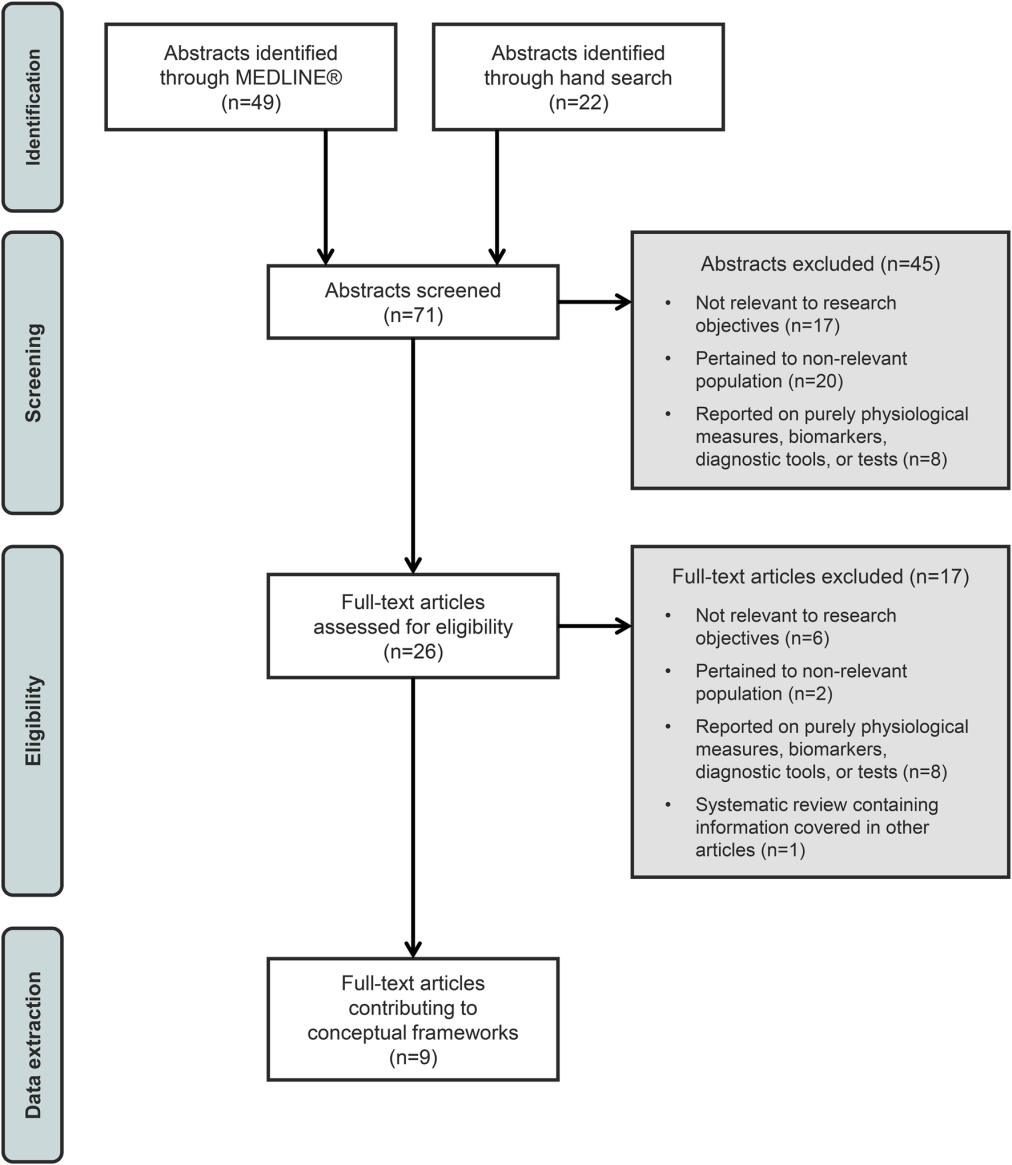
Diagram for article identification and selection

The literature review via MEDLINE^®^ was conducted in October 2019 and updated in 2024. Initially, 49 abstracts were identified and reviewed after removing duplicates. Most abstracts did not meet the eligibility criteria for full-text review, resulting in only four abstracts being prioritized for the next phase of developing preliminary conceptual frameworks. After full-text review, one article was excluded for only including information on asymptomatic females with OTCD, leaving three articles identified via MEDLINE^®^ contributing to the preliminary conceptual frameworks. An additional 22 articles were identified via hand search and six contributed to the development of the preliminary conceptual frameworks. During the full-text review of available literature, 36 distinct patient-reported signs and symptoms and seven distinct patient-reported impacts of OTCD were identified. See Fig. 3 for a diagram detailing the article selection process, including specific reasons for article exclusion.

The literature review was updated in October 2024 to identify more recent literature (six articles) to contribute to the discussion of patient-reported experiences with OTCD; however, the results were not used to modify the conceptual frameworks as no new themes emerged that would alter the interpretation of the initial literature review results.

Additionally, no OTCD-specific or UCD-specific COA instruments were identified in the literature. The generic Pediatric Quality of Life Inventory™ (PedsQL) and 36-item Short Form Survey (SF-36) were found to have been used in previous UCD research; however, the literature search did not yield any disease-specific COAs.

### KOL interviews

Three KOLs were interviewed, including a physician/laboratory scientist, medical geneticist, and biochemical geneticist who had between 20 and 45 years of experience treating patients with UCDs. One KOL reported seeing 40–50 patients with OTCD over the course of his career, while the other two reported that they most commonly treated patients with OTCD.

The three KOLs were interviewed about signs and symptoms of UCDs in general rather than those specific to OTCD. However, they indicated that the signs and symptoms generally do not differ among UCDs when asked to confirm whether the signs and symptoms they mentioned were relevant to OTCD. All three KOLs (100.0%) described that patients with UCDs experience abnormal or loss of appetite, ataxia, cognitive issues, coma, confusion, fatigue or lethargy, and vomiting. Additionally, executive functioning difficulties mentioned included difficulty starting tasks, maintaining focus, self-monitoring (i.e. regulating emotions and behaviors), problems with organization, and planning. Two KOLs (66.7%) reported apnea, liver issues, and nausea. The following UCD symptoms were mentioned by one clinician (33.3%): changes in behavior or mental status, dizziness, hypothermia, migraine, seizure, spasticity, speech difficulties, stomach pain or discomfort, and transient blindness.

Regarding hyperammonemia events, the KOLs emphasized how symptoms may progress, with hyperammonemia events often starting with symptoms like nausea, vomiting and dizziness, progressing to stupor and coma if untreated, and potentially resulting in death. Confusion and vomiting were mentioned as early signs at the onset of hyperammonemia events. All three KOLs (100.0%) reported ataxia, coma, and death as possible during the events, with two clinicians (66.7%) additionally reporting nausea, lethargy and vomiting. The following were noted as potential post-event complications: cognitive issues, ataxia, spasticity, and transient blindness.

When asked about the most important signs and symptoms to treat in UCDs, each clinician (100.0%) communicated a focus on prevention. One KOL (33.3%) stated that recognition of early signs of hyperammonemia events (e.g. neurological symptoms and behavioral changes) is the best method for treating UCD symptoms.

The KOLs were asked to report impacts of UCDs and only those relevant to OTCD are described here. Several impacts around dietary restrictions were identified as a protein-restricted diet is necessary to prevent hyperammonemia events, with non-compliance potentially leading to death. Another common impact was the therapeutic burden to patients as UCDs involve a strict and costly therapeutic regimen. Emotional and social impacts included low self-esteem, anxiety, and strained relationships among patients and caregivers; further, energy expenditure was identified as important given the potential for exertive physical activity to contribute to hyperammonemia events. Finally, post-hyperammonemia event impacts were reported to include cognitive impairments affecting walking, fine motor skills, school/work performance, and sleep.

The KOLs offered diverse opinions on the most critical impacts to address. Two (66.7%) focused on preventing hyperammonemia events and one KOL (33.3%) focused on diet management. One KOL (33.3%) added that minimizing neurological impacts was the most important impact to address. Regarding the most important concepts to include in HRQoL measures, two KOLs (66.7%) mentioned dietary restrictions and one KOL (33.3%) specified general quality of life, depression, and anxiety each as specific concepts to measure.

### Initial concept elicitation interviews

Two adult participants with OTCD participated in an initial round of interviews. The small sample size reflects the rarity of OTCD and aligns with similar studies on rare diseases that often face challenges in recruiting large numbers of participants due to the limited population affected by these conditions [19]. Additionally, individuals with OTCD can struggle with executive function, making it potentially difficult for them to make and keep appointments such as interviews, which aligns with the KOLs reporting patient challenges with organization and planning.

A combined total of 16 symptoms of OTCD were reported by the patients. Loss of appetite, dizziness, headaches, nausea, and vomiting were reported by the two patients (100.0%), with both experiencing nausea 2–3 times per month but having significantly different vomiting frequencies; one participant (50.0%) reported vomiting due to OTCD only three times total, all during hyperammonemia events, while the other participant (50.0%) reported vomiting every few months over the past nine years and previously vomiting weekly. Physical symptoms reported by one patient each (50.0%) included hot/cold sensations, hyperammonemia events, sudden change in feeling (e.g. feeling disoriented), lethargy, stomach pain, diarrhea, and tiredness. Additionally, cognitive symptoms reported by one patient each (50.0%) included ataxia, difficulty paying attention, poor spatial understanding, and confusion. When asked which signs and symptoms were the most important to treat, one patient (50.0%) prioritized loss of appetite and difficulty focusing/paying attention, while the other patient (50.0%) prioritized vomiting.

The two patients reported six impacts across diet, emotional, and social domains. Both participants (100.0%) reported dietary restrictions and explained the challenges of avoiding protein in their meals. For emotional impacts, both participants (100.0%) reported feeling embarrassed due to dietary restrictions as well as having fear around hyperammonemia events. For social impacts, both patients (100.0%) reported unwanted attention linked to their dietary restrictions and medication regimen. One patient (50.0%) reported the additional impacts of difficulty tracking diet and feeling different from others. When asked which impacts were the most important to improve, one patient (50.0%) identified difficulty tracking diet and the other (50.0%) prioritized feeling different from others. Preliminary conceptual frameworks were developed based on these results. Additional interviews were needed to confirm or modify the proposed concepts prior to finalizing the conceptual frameworks.

### Additional concept elicitation interviews

To supplement the initial two concept elicitation interviews and explore the relevance of the signs, symptoms, and impacts of OTCD included in the preliminary conceptual frameworks, additional interviews were conducted with 10 participants, for a total of 12 participants: seven adult patients (*n* = 7), two adolescent patients (*n* = 2), two caregivers (*n* = 2) and one pediatric patient (*n* = 1). A summary of demographic and health information for the patients is found in Table 1.

**Table 1:** Demographic and health information for adult, adolescent, and pediatric patient participants

Characteristic	Adult patients (n = 7) n (%)	Adolescent patients (n = 2) n (%)	Pediatric patient (n = 1) n (%)	Total patient sample (N = 10) n (%)
**Age (in years)**				
Minimum - maximum	26–71	15–17	8	8–71
Mean (standard deviation)	43.7 (17.4)	16.0 (1.4)	8.0 (0.0)	35.0 (20.5)
**Sex**				
Female	7/7 (100.0%)	2/2 (100.0%)	1/1 (100.0%)	10/10 (100.0%)
**Race (all that apply selected)**^**a**^				
White	7/7 (100.0%)	N/A	N/A	N/A
**Ethnicity**^**a**^				
Not Hispanic/Latino(a)	6/7 (85.7%)	N/A	N/A	N/A
Hispanic/Latino(a)	1/7 (14.3%)	N/A	N/A	N/A
**General health status**^**b**^				
Excellent	0/7 (0.0%)	0/2 (0.0%)	0/1 (0.0%)	0/10 (0.0%)
Very good	2/7 (28.6%)	0/2 (0.0%)	1/1 (100.0%)	3/10 (30.0%)
Good	5/7 (71.4%)	2/2 (100.0%)	0/1 (0.0%)	7/10 (70.0%)
Fair	0/7 (0.0%)	0/2 (0.0%)	0/1 (0.0%)	0/10 (0.0%)
Poor	0/7 (0.0%)	0/2 (0.0%)	0/1 (0.0%)	0/10 (0.0%)
**Currently being treated with a restricted diet or ammonia scavenger medication**				
Yes	7/7 (100.0%)	2/2 (100.0%)	1/1 (100.0%)	10/10 (100.0%)
**In addition to ammonia scavengers, taking additional medication for OTCD**				
Yes	4/7 (57.1%)	1/2 (50.0%)	1/1 (100.0%)	6/10 (60.0%)
No	3/7 (42.9%)	1/2 (50.0%)	0/1 (0.0%)	4/10 (40.0%)

^a^Race ethnicity data were not supplied for adolescent and pediatric patients

^b^General health status’ was a bespoke item included in this study’s Demographic and Health Information Form completed by adult participants or by the caregivers on behalf of adolescent participants prior to their scheduled interviews. The item asks, “How would you describe your health in general?” with response options of “Excellent, Very good, Good, Fair, Poor.”

Of the 12 total participants (2 participants from the initial interviews and 10 participants from the additional interviews), the frequency of hyperammonemia events reported ranged from none to “innumerable” due to “improper manage[ment]” for 13 years. Similarly, the number of hospitalizations due to OTCD ranged from none to “hundreds”. Specifically, participants reported the following hyperammonemia event frequency: 0 events (16.7%; one adolescent patient and their caregiver), 1 event (16.7%; two adult patients), 2 events (16.7%; two adult patients), 3 events (8.3%; one adult patient), 4 events (16.7%; one adult patient and the caregiver of the pediatric patient), 40 events (8.3%; one adolescent patient), and “innumerable” (8.3%; one adult patient). The pediatric patient was not able to report the number of hyperammonemia events they had experienced to date. Participants commonly reported hyperammonaemia events (50.0%) as a symptom and the fear of hyperammonemia events (58.3%) as an impact. One participant described the severity of their hyperammonaemia events, explaining, “I had my first main [hyperammonaemia event] at diagnosis. That was the worst one, because that one put me in a coma for eight days” (01–07-adult patient).

**Fig. 4:**
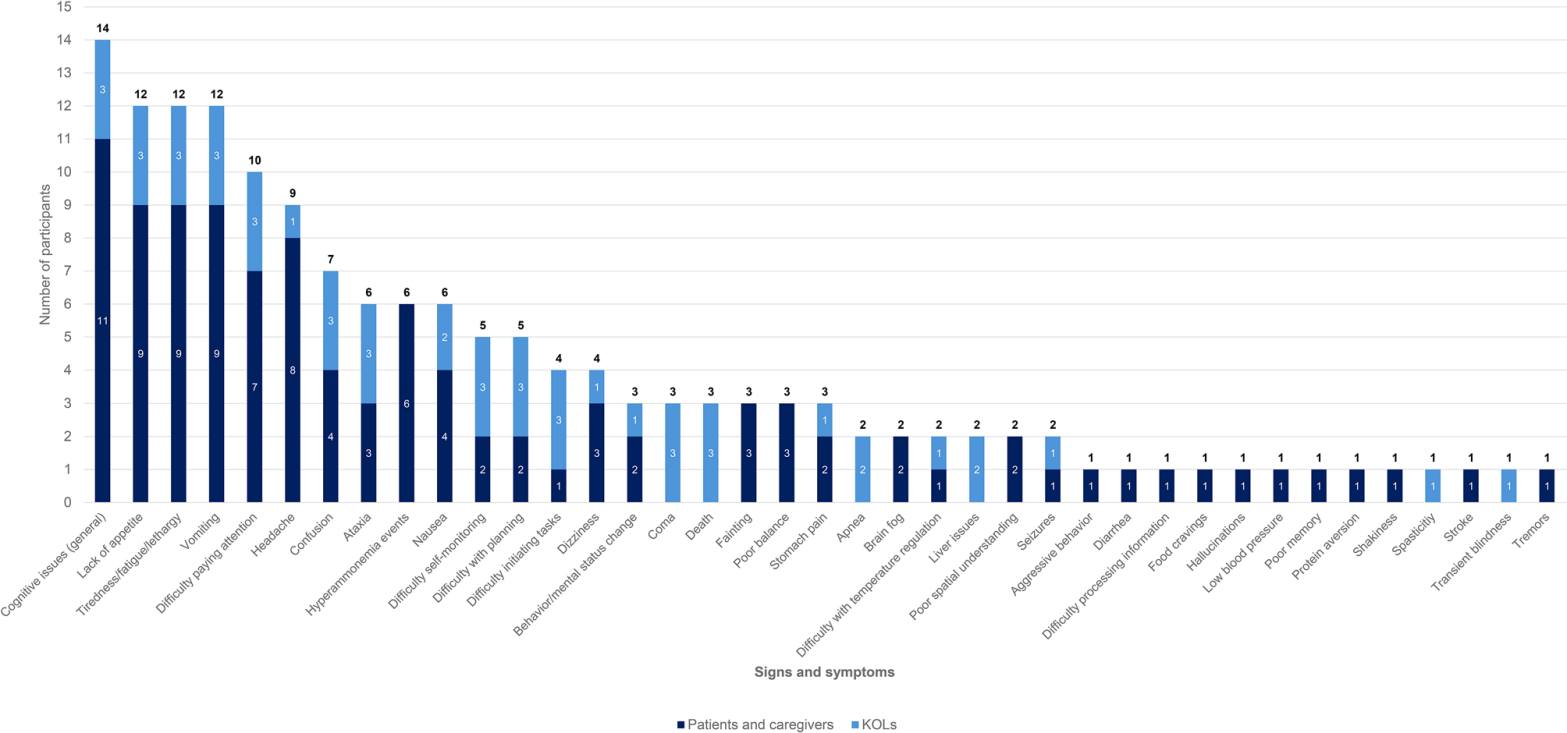
Patient/caregiver- and KOL-reported signs and symptoms of OTCD

**Fig. 5:**
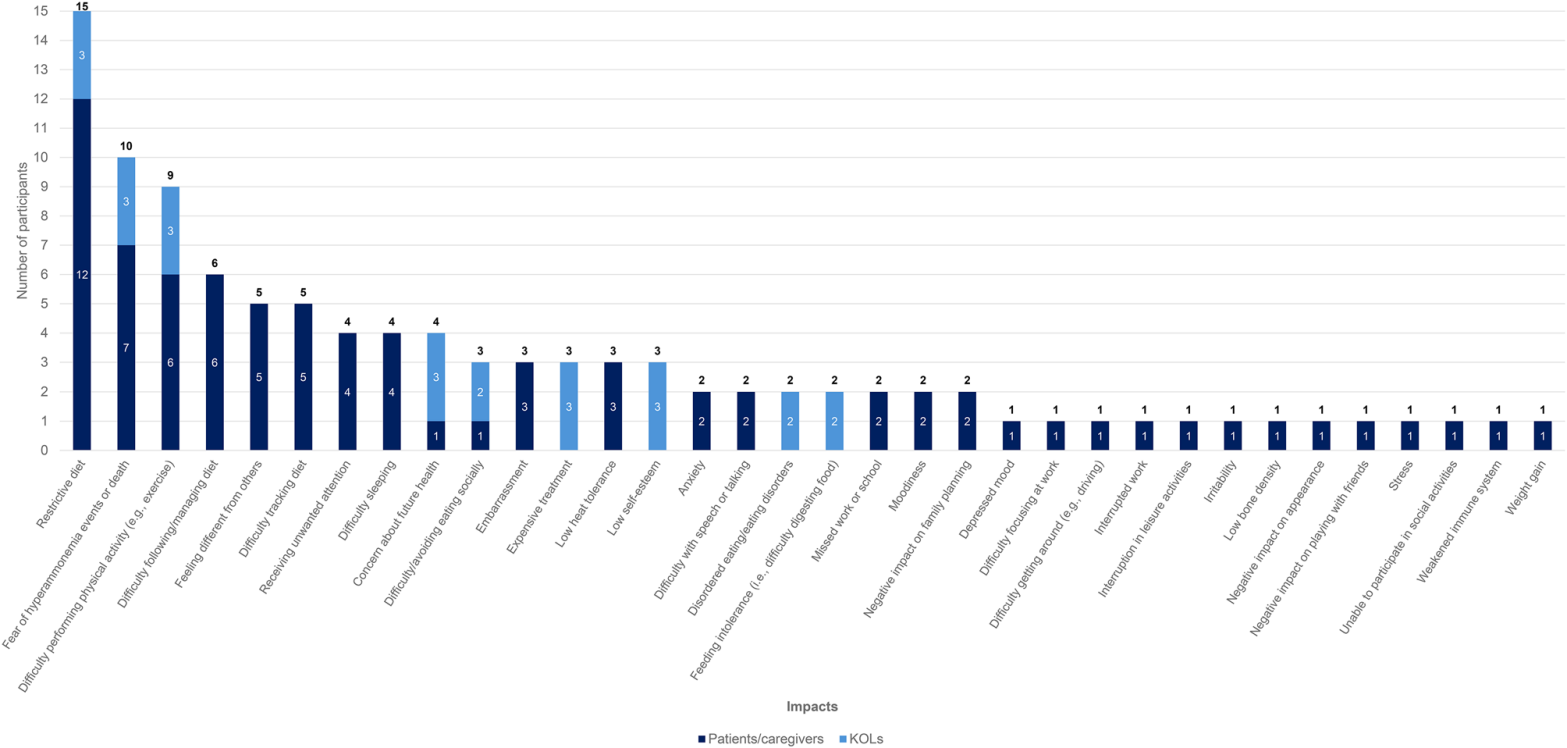
Patient/caregiver- and KOL-reported impacts of OTCD

In terms of symptoms and impacts of OTCD, nine participants (75.0%) reported vomiting and loss of appetite as key symptoms of OTCD, followed by headache (66.7%), difficulty focusing/paying attention (58.3%), and tiredness (50.0%). A participant described the disruptive onset of their vomiting by stating, “I remember leaning against a wall, because I thought people are going to think I’m drunk or doped up, because I couldn’t walk … I started vomiting and got home and went to bed and stayed in bed for two days” (01–01-adolescent patient). Primary impacts of OTCD reported were restricted diet (100.0%), difficulty performing physical activity (e.g. exercise) (50.0%), difficulty adhering to diet (50.0%) and feeling different from others (41.7%). One participant described the constant struggle of the restricted diet, explaining, “I low-key love hot wings, but I can’t have them. And when people eat them in front of me, it’s just like teasing … It’s hard to – having to manage, knowing like I really love to eat this. If I eat this, this could be very- this could be bad” (01–07-adolescent patient). Figures 4 and 5 summarize the patient/caregiver- and KOL-reported symptoms and impacts of OTCD via stacked bar graphs in order of overall frequency of report. Symptoms and impacts of OTCD reported by KOLs were included in these figures to highlight the overlap in key symptoms identified by both samples, such as neurocognitive issues, loss of appetite, tiredness, and vomiting, as well as key impacts like restrictive diet, fear of hyperammonemia events, and difficulty performing physical activity.

### Development of final conceptual frameworks for PROs

**Fig. 6:**
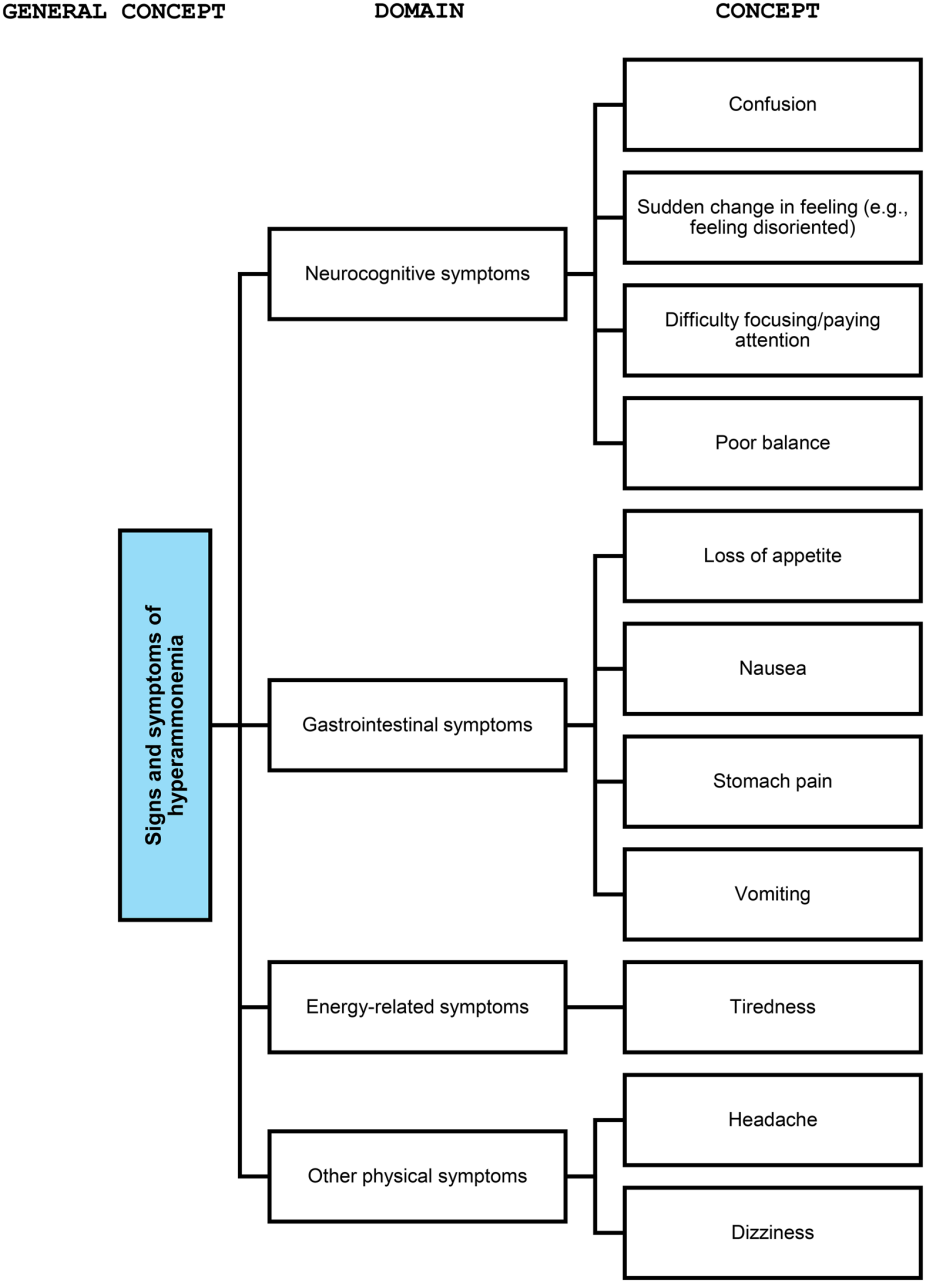
Final conceptual framework for signs and symptoms of OTCD

A final conceptual framework for signs and symptoms of OTCD was developed through the literature review in conjunction with the findings of the KOL and patient interviews and included 11 essential item-level concepts that were organized into four domains: neurocognitive, gastrointestinal, energy-related, and other physical symptoms. Some concepts were excluded if they were only found in literature, not reportable by patients (an important consideration for PRO measure selection and development), overlapped significantly with other concepts, or were only relevant in severe cases. A summary of the included signs and symptoms can be found in Table 2, which specifies the source for each symptom (i.e. literature review, KOL interviews, or patient/caregiver interviews), the frequency of report per source (i.e. number of articles, KOLs, and/or patients/caregivers reporting each concept) and has accompanying quotes from interviews. The conceptual framework for signs and symptoms of OTCD by domain is presented in Fig. 6 and has been finalized based on the data gathered through in this study. This conceptual framework is both reflective and formative since the signs and symptoms are indicators of OTCD as well as defining characteristics of OTCD (i.e. form the OTCD symptom experience).

**Table 2:** Summary of identified signs, symptoms, and impacts of OTCD

Concept	Source	n (%)	Exemplary quotes
**Signs and symptoms**			
*Neurocognitive*			
Confusion	• Literature (clinician- or patient-reported)	2/9 (22.2%)	Adult patient: Well, you know when your ammonia’s going up, because you feel lethargic and a little out of it. … Confusion. … It’s a mental thing. … I’ll try to focus, and I say to her, if you see me staring at you, you know that this is happening. That’s what’s happening to me and just be aware that if it gets any worse, you have to do something.
	• KOL interviews	3/3 (100.0%)	
	• Patient and caregiver interviews	4/12 (33.3%)	
Sudden change in feeling (e.g. feeling disoriented)	• KOL interviews	1/3 (33.3%)	Adult patient: And I walked out of [doctor’s office], and I remember leaning against a wall, because I thought people are going to think I’m drunk or doped up, because I couldn’t walk. … I could – it was like being totally, totally, totally drunk. That’s the only way I can say it. So drunk you can’t walk. … And I looked at my eyes and my eye – you get a glassy, drunken look in your eyes. … and I knew before – they knew what my level was, before any of the symptoms showed up, I knew what was going to happen, because I could – I looked at my eyes and … It’s that your eyes look like a drunk’s.
	• Patient and caregiver interviews	2/12 (16.7%)	
Difficulty focusing/paying attention	KOL interviews	3/3 (100.0%)	Adult patient: I’ll just completely zone out, and people will be talking to me, and I won’t realize it. … A couple times a day. … A couple minutes, I guess. I don’t really zone out completely, but I will notice it.
	• Patient and caregiver interviews	7/12 (58.3%)	
Poor balance	• Literature (clinician- or patient-reported)	1/9 (11.1%)	KOL: Oh, they – yeah, that’s part of a whole neurologic picture. When you cramp up the brain, you get al.l of these symptoms. You get unsteady. You can’t stand. You fall …
	• KOL interviews	3/3 (100.0%)	
	• Patient and caregiver interviews	3/12 (25.0%)	
*Gastrointestinal*			
Loss of appetite	• Literature (clinician- or patient-reported)	1/9 (11.1%)	Adult patient: I can go almost like the whole day without eating sometimes. I’m just not hungry.
	• KOL interviews	2/3 (66.7%)	
	• Patient and caregiver interviews	9/12 (75.0%)	
Nausea	• KOL interviews	2/3 (66.7%)	Adult patient: Well, there’s – sometimes there’s nausea … Maybe three times a month. … The last time I had it really bad, I forgot take my night dose of Ravicti, and the next morning when I woke up, I thought uh-oh, I’m in trouble and that lasted until I took the next Ravicti and waited like an hour or so for it to kick in.
	• Patient and caregiver interviews	4/12 (33.3%)	
Stomach pain	• KOL interviews	1/3 (33.3%)	Adult patient: Kind of scary, because I didn’t know what was wrong with me. I couldn’t really get out of bed. And when I did, it was to the bathroom. I had really bad stomach pains.
	• Patient and caregiver interviews	2/12 (16.7%)	
Vomiting	• Literature (clinician- or patient-reported)	4/9 (44.4%)	Adult patient: We got in the car for – about 10 minutes later I said pull over, because I’m going to throw up and then I started vomiting and got home and went to bed and stayed in bed for two days. … Because the ammonia went up. … It only happens – if you – if someone having a urea cycle disorder starts vomiting, you’re in trouble and you need to get to the emergency room now. That’s a sign.
	• KOL interviews	3/3 (100.0%)	
	• Patient and caregiver interviews	9/12 (75.0%)	
*Energy-related*			
Tiredness	• Literature (clinician- or patient-reported)	2/9 (22.2%)	Adult patient: And sometimes during the day, I become tired and/or like a little out of it and that usually occurs when I’m within 45 minutes of my dose of Ravicti. … But sometimes you feel like OK, I’m sitting here, and I could fall asleep any minute.
	• KOL interviews	3/3 (100.0%)	
	• Patient and caregiver interviews	6/12 (50.0%)	
*Other physical*			
Headache	• KOL interviews	1/3 (33.3%)	Adult patient: I – sometimes I’ll have really bad headaches most of the day. … They’re mostly in my forehead area. They get really pounding. And they hurt really bad. … with the headaches – I’m assuming it’s related to that [OTCD] anyway – and then I get really nauseous.
	• Patient and caregiver interviews	8/12 (66.7%)	
Dizziness	• KOL interviews	1/3 (33.3%)	Adult patient: I’ll get really dizzy or really hot or – and then I’ll get really cold. It just depends. I’m assuming that would be like an OTC attack or whatever you would want to call it, but I’m not sure. … I’ll get dizzy and I’ll end up laying on the bathroom floor, and I won’t remember how I got there.
	• Patient and caregiver interviews	3/12 (25.0%)	
**Impacts**			
*Diet-related*			
Restricted diet	• Literature (clinician- or patient-reported)	1/9 (11.1%)	Adult patient: I just don’t have a normal diet. That’s the bottom line. I don’t have a normal diet. They figure I have about 30% of the enzyme, so I’m on the low end. My normal breakfast is usually two chocolate chip cookies and coffee with – black with sugar. I don’t eat lunch and dinner is pasta or some kind of vegetable plate. … I typically consume they figure about 15 grams of protein a day and I doubt that I get even that … I’ve always avoided protein, so it’s normal for me …
	• KOL interviews	3/3 (100.0%)	
	• Patient and caregiver interviews	12/12 (100.0%)	
Difficulty adhering to diet	• KOL interviews	3/3 (100.0%)	Adult patient: I mean, it’s hard to keep track of my protein, but that’s the only really thing that I have difficulty managing. … I mean, I’d like to learn more. But I don’t really know how to track my protein, so that kind of bothers me.
	• Patient and caregiver interviews	6/12 (50.0%)	
*Emotional*			
Fear of hyperammonemia events	• Patient and caregiver interviews	7/12 (58.3%)	Adult patient: My husband died three years ago, so now I’m in the house alone. My son died in 1996, because he had full-blown neonatal onset OTC. Boys don’t live. And I said to the doctor at – Dr. [last name] at [medical practice] – I said I’m alone. What do I do if I think my ammonia’s going up? And he gave me a letter … And it tells the medications I’m on, who to call and where – what should be done and what hospital I should be taken to. … And I said to him, like if I – if it’s 2:00 in the morning, what do I do? He said call 911. I said well, there’s no place around here that’s going to know what to do and he gave me – and it’s on here – a document that says – I should be taken to [hospital], which is not close by, treated there and then sent to him in [city], in the city. And that’s a fear.
Feeling different from others	• Patient and caregiver interviews	5/12 (41.7%)	Adult patient: I guess it was two weeks ago, we went to a ladies’ luncheon and my table was one of the last called up. It was a buffet. The only thing left were sandwiches, corned beef, roast beef and something else and salad and they said – I got back to the – my table and they said to me that’s all you’re eating is salad? And you’re five again, it’s embarrassing. … And people don’t get it and you don’t want to go into this whole big deal about why.
Feeling embarrassed	• Patient and caregiver interviews	3/12 (25.0%)	Adult patient: I had to take medicine twice a day at school. So it – to me, that was kind of embarrassing to have to get up at a certain time of the day and go to the nurse’s office and take medicine. And – I don’t know, kids just look at you a certain way.
Low self-esteem	• KOL interviews	3/3 (100.0%)	KOL: Q: Would low self-esteem be an impact that you’ve encountered? A: It’s going to depend on – the answer’s probably. I don’t know that I’ve ever thought much about that or in reading about it, whether I’ve noticed that, but clearly, imagine the teenager who’s that different and who’s on a special diet. They’re certainly going to be self-conscious and so without knowing the answer, I would say yes.
*Physical*			
Difficulty performing physical activity	• KOL interviews	1/3 (33.3%)	Adult patient: It stops me from exercising as much as I would like to, I would say. … Well, I took one exercise course. I was take – it was called Country Heat. It’s a Beach Body class here at the rec center in town and it affected by numbers [ammonia levels] enough that they had to up my Ravicti and that was one hour a week. … So I can’t – some people work out fine – so I stick to things like walking.
	• Patient and caregiver interviews	6/12 (50.0%)	
*Sleep*			
Difficulty falling asleep	• Patient and caregiver interviews	4/12 (33.3%)	Adult patient: It’s the OTC acting – making me restless. … In other words, I can’t sleep. Sometimes I’m not falling asleep until 1:00, 2:00. Sometimes I don’t sleep at all. … it’s like oh God, I want to sleep. I can feel my eyes heavy, but it’s like nothing.
*Social*			
Receiving unwanted attention	• Patient and caregiver interviews	4/12 (33.3%)	Adult patient: I take Ravicti, which can be embarrassing, because – have you seen people take Ravicti? … You have a little syringe and no matter where you are, you have to take out – this is the Ravicti in the bottle, and you have to measure it out in the syringe, and no matter where you are, you have to look around and shoot it in your mouth. It’s like what’s she doing?

**Fig. 7:**
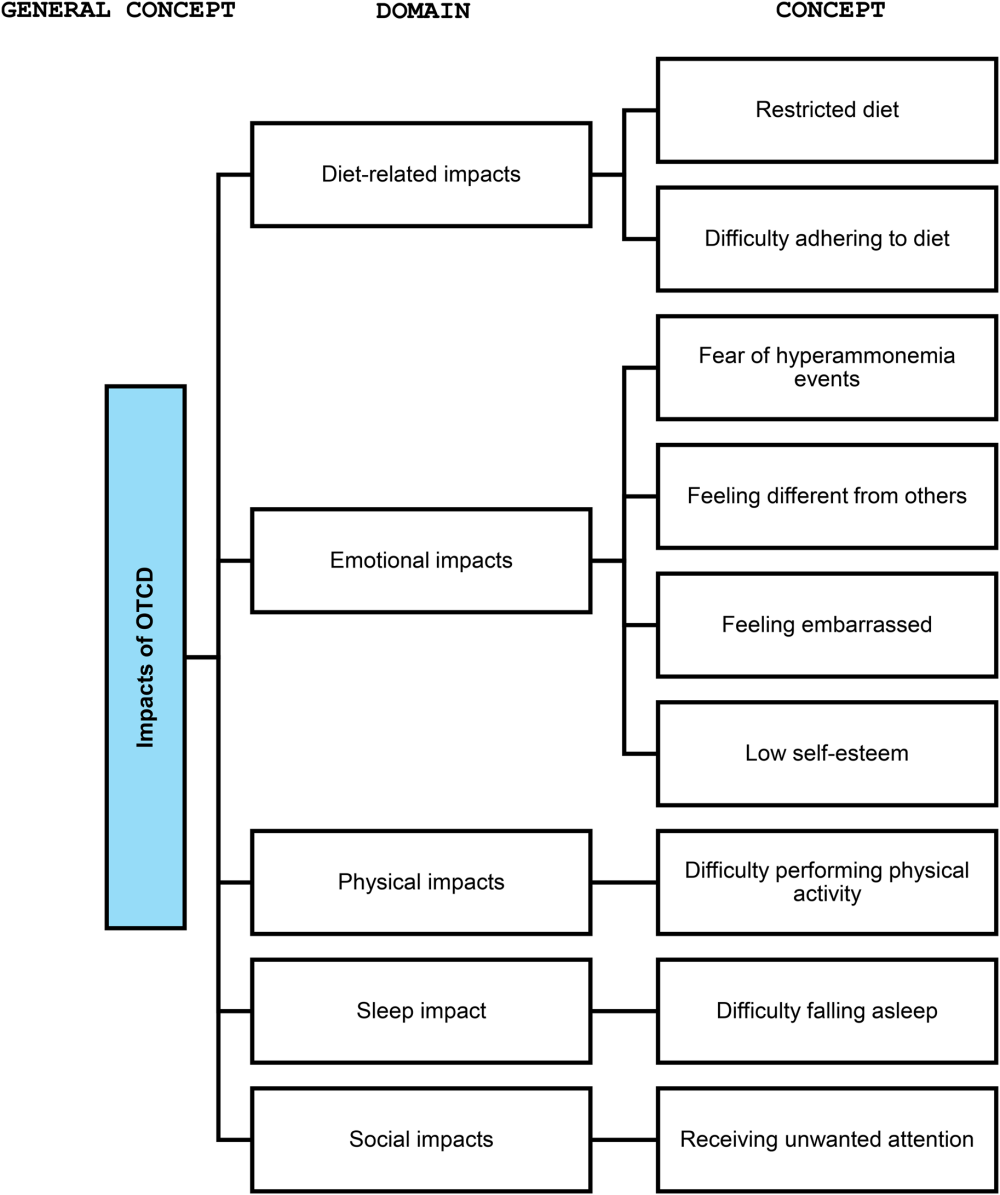
Final conceptual framework for impacts of OTCD

A conceptual framework for the impacts related to OTCD was also developed. This included nine item-level concepts that were grouped into five domains: diet-related, emotional, physical, sleep, and social impacts. Inclusion criteria differed for impacts as fewer were found across the sources, so concepts appearing in only one source may have been considered if they seemed to be of interest to patients and future investigations. Concepts were excluded if they necessitated a clinical diagnosis or could be more effectively evaluated through other methods, were identified in only one source and were not of interest to patients and future investigations, were unlikely to change in a clinical trial, or pertained to treatment satisfaction. A summary of the included impacts is found in Table 2, which shows the source of the information for each impact, frequency of report by source, and accompanying quotes from the interviews. The conceptual framework for impacts of OTCD by domain is presented in Fig. 7 and represents the updated version based on the findings from this study.

## Discussion

The objective of this research, to develop conceptual frameworks to characterize humanistic burden and to inform the identification or development of COAs for OTCD, was pursued through a literature review, KOL interviews, and concept elicitation interviews with patients with OTCD ages 8 and older and caregivers. These draft conceptual frameworks were tested and refined through the multiple rounds of interviews and are recommended to undergo further testing in mixed concept elicitation and cognitive debriefing interviews with more patients.

The process of triangulating data from multiple sources – literature reviews, KOL insights and patient experiences – not only enhances the robustness of the findings but also strengthens the study’s overall credibility. This methodological approach ensures that the conceptual frameworks are well-founded and effectively capture the lived experiences of individuals with OTCD, thereby supporting the future development of reliable and valid COAs.

The results from the interviews allow patient voices to be a primary contributor to the conceptual frameworks. Findings from the literature review highlight how the patient perspective on living with OTCD is lacking as much of the current research falls under UCDs as a whole (e.g. Longitudinal Study of Urea Cycle Disorders [20]). Clinician survey research in UCD management found that most clinicians rate the symptoms of UCD as very/extremely bothersome to the daily lives of patients and their families [21]. The insights from this study, focused specifically on OTCD, underscore the unique challenges patients and their caregivers face, providing valuable context for clinical care and the development of targeted intervention.

As is common with other rare diseases, the presentation of OTCD is heterogeneous and often serious and potentially life threatening. Early signs and symptoms of OTCD such as confusion, nausea, vomiting, and dizziness may be a precursor to a hyperammonemia event, which could lead to coma and possibly death. Even when not facing a hyperammonemia event, patients experience significant neurocognitive, gastrointestinal, and physical decrements that limit HRQoL. Although caregiver impact was not specifically investigated in this study, previous research suggests that caring for a child or family member with a UCD can have a negative impact on HRQoL.[21]

In common with many diseases with high prevalence rates, there are a host of emotional and social impacts that arise due to the disease. What is somewhat atypical for OTCD and UCDs in general is that standard of care management is associated with significant burden. Dietary restrictions can be difficult to maintain and track, with lack of adherence causing serious consequences. In addition, dietary management can cause embarrassment, unwanted attention, and make patients with OTCD feel different from their peers.

Comparisons to other rare diseases that rely heavily on dietary management are informative to the OTCD population. In glycogen storage disease type I (GSD1a), where dietary management is the standard of care, like in OTCD, difficulty with independent functioning has been reported in the literature due to the complicated medical and dietary regimen that requires parental involvement [22]. This research is consistent in that one of the most important HRQoL domains identified in the interviews was restriction of diet and adhering to a specific diet.

Limitations of this study are largely related to recruitment challenges. Since the study targeted patients with OTCD, a rare disease, there was difficulty in finding an adequate number of participants for both rounds of concept elicitation interviews to inform the frameworks from the patient perspective. However, the small sample size was balanced with the KOL interviews representing 20–45 years of experience from experts who routinely see patients with OTCD in their practice. Another limitation pertains to participant self-selection bias since those with more severe symptoms or those more engaged in their health care and patient advocacy may have chosen to participate. Moreover, the limited number of participants in the study resulted in patient and caregiver data from only White female participants, which limits the generalizability of the findings. This is particularly important because OTCD tends to manifest differently across genders; males typically exhibit more severe symptoms, while females may experience milder symptoms due to the X-linked pattern of inheritance [2]. As a result, drawing conclusions applicable to the broader disease population is difficult due to these demographic limitations. Future research can overcome sample size challenges by partnering with patient advocacy groups, utilizing social media for outreach, and leveraging rare disease patient registries. Further, pulling from clinical trial populations for qualitative interviews could aid with recruitment and ensure a global perspective is captured, thereby mitigating self-selection bias.

Despite the study being conducted in the US, the conceptual frameworks have significant global applicability, as OTCD occurs worldwide and patients encounter similar challenges [23]. By providing a structured approach to assess the specific burdens associated with OTCD, these conceptual frameworks can inform the development of COA measures and interventions that are applicable in diverse healthcare settings, which can improve patient care and HRQoL globally by standardizing the understanding and management of OTCD.

The practical application of these findings in clinical care includes developing COAs specifically tailored to capture the nuances of OTCDs impact on patients’ lives. While generic COAs like the Pediatric Quality of Life Inventory™ (PedsQL™) or 36-Item Short Form Survey (SF-36) may capture general physical, psychosocial, and emotional functioning in patients with OTCD [12, 13], disease-specific measures could be more sensitive to detection and quantification of small changes that are unique and/or important to the OTCD population [15]. Disease-specific COAs allow for a more precise and relevant assessment of the specific symptoms, challenges and impacts experienced by patients, which generic tools might overlook as they are purposely designed to be applicable to diverse populations and conditions. There is a dearth of information on HRQoL impacts in OTCD, and academic investigations in UCD have shown mixed results of the impact on HRQoL [13] – possibly due to the use of generic COAs such as the SF-36 and PedsQL in studies. Instead, disease-specific COAs could be incorporated into clinical trials to assess the efficacy of new treatments and interventions more accurately [15]. Further, disease-specific COAs could become standard across both trials and clinical settings to allow for larger data sets for comparison and understanding of this patient population to guide health/treatment policy for patients and clinicians. This targeted approach can inform clinicians about the most significant areas of patient burden, allowing for more personalized and effective treatment plans that address both medical and psychosocial needs. In order to develop new disease-specific COAs, the conceptual frameworks developed in this study should undergo additional testing in mixed concept elicitation and cognitive debriefing interviews with more OTCD patients (or their caregivers), after which draft COAs can be developed through collaboration with clinical experts, debriefed with additional OTCD patients, refined, and validated through psychometric testing. As of yet, the potential lack of sensitivity of generic COAs in the OTCD population has not been fully researched, so future research should explicitly compare the performance of generic measures with OTCD-specific measures (once developed) in the target population to further support the validity and use of new COAs.

Future research guided by the findings of this study can also help to generate evidence-informed policymaking via patient/stakeholder involvement. In medical research, patients are no longer strictly research participants but also experts in their own lived experience, including the symptoms and impacts of their condition that affect them the most; therefore, their unique insights such as those elicited in this study can serve to inform clinical trial designs, endpoint strategies, and relevant research policies and practices [24, 25]. Additionally, the frameworks can direct policy by highlighting the need for more comprehensive support systems for individuals with OTCD and their caregivers. Policymakers can use these insights to advocate for increased funding for research, improved access to specialized care and the development of educational programs that raise awareness about the disease and its management.

Ultimately, the conceptual frameworks presented here offer the possibility of assessing specific disease-related burden through development of OTCD-specific COAs that may be more sensitive to measuring burden of disease and changes over time compared to generic measures currently being used to assess HRQoL. Future tailored COAs may enhance clinical trials and interventions, leading to more effective management strategies and ultimately provide better outcomes for patients with OTCD. By integrating these frameworks into clinical practice, healthcare providers can better address the comprehensive needs of OTCD patients to improve patient satisfaction and quality of life.

## Conclusions

This research represents a novel contribution to the knowledge base of OTCD in which patient-reported data have not yet been widely examined or shared. Overall, concepts identified in the literature review and KOL interviews were also found to be relevant to the participants that completed the concept elicitation interviews in that they experienced them due to their OTCD. The information gathered from the qualitative interviews addresses the gap in literature incorporating the patient perspective of OTCD and lays the foundation for future development of accurately tailored COAs.

The most frequently reported symptoms, such as hyperammonemia events and loss of appetite, along with impacts like challenges in tracking diet and managing dietary restrictions, are all addressed in some form within the preliminary frameworks. This suggests that the conceptual frameworks encompass the concepts most important to patients, as advised by the FDA guidance on human gene therapy for rare diseases [15].

The next step for this work is to assess the content validity of the conceptual frameworks and the development of COAs specifically for use in the OTCD population. To guide this process, the roadmap below outlines the key next steps necessary to advance this research effectively.
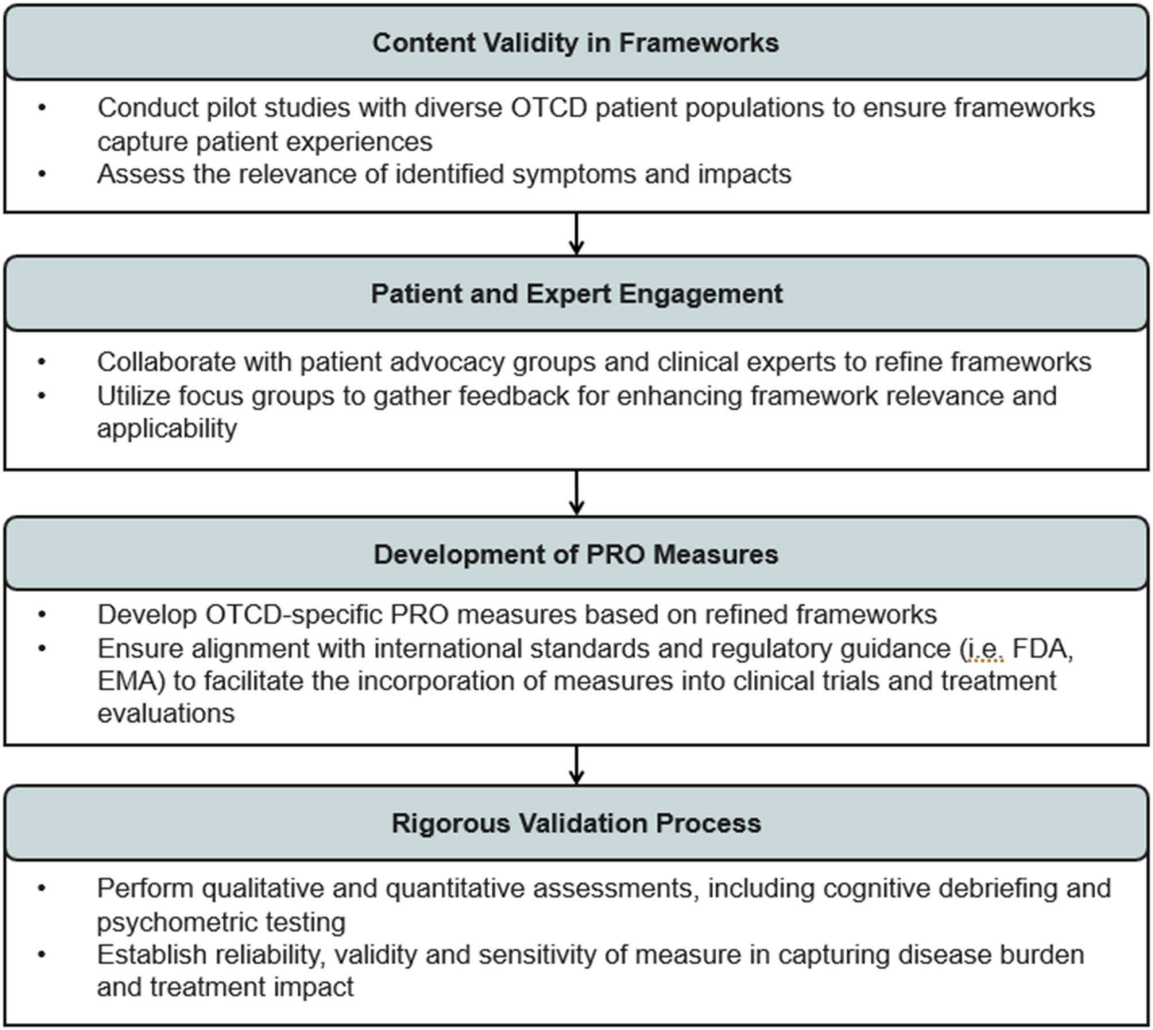


By following this roadmap, the understanding and management of OTCD, contributing to the development of targeted interventions that improve the quality of life for individuals affected by this rare condition, will be enhanced.

## Electronic supplementary material

Below is the link to the electronic supplementary material.

Supplementary Material 1

## Data Availability

The datasets generated and/or analysed during the current study are not publicly available due to study participant privacy restrictions but are available from the corresponding author on reasonable request.

## Abbreviations

ARGD: Arginine-1 deficiency
ASLD: Adenylosuccinate lyase deficiency
COA: Clinical outcome assessment
ClinRO: Clinician-reported outcome
CPS1D: Carbamoyl phosphate synthetase 1 deficiency
EMA: European Medicines Agency
FDA: Food and Drug Administration
GSD1a: Glycogen storage disease type I
HHH: Hyperornithinemia-hyperammonemia-homocitrullinuria
HRQoL: Health-related quality of life
IRB: Institutional review board
KOL: Key opinion leader
NAGSD: N-acetylglutamate synthetase deficiency
NUCDF: National Urea Cycle Disorders Foundation
ObsRO: Observer-reported outcome
ORNT1D: Ornithine transporter 1 deficiency
OTCD: Ornithine transcarbamylase deficiency
PedsQL: Pediatric Quality of Life Inventory™
PerfO: Performance outcome
PRO: Patient-reported outcome
QOL: Quality of life
SF-36: 36-item Short Form Survey
UCD: Urea cycle disorder
WCG: Western Copernicus Group
