# Supplementary material for: Development of patient-centric conceptual frameworks for symptoms and impacts of ornithine transcarbamylase deficiency (OTCD)
Source: J Patient Rep Outcomes. 2025 Oct 22;9:123. doi: 10.1186/s41687-025-00939-5 (PMC12545949; doi:10.1186/s41687-025-00939-5)
Supplement: Supplementary file 1 — Supplementary Material 1 [file 41687_2025_939_MOESM1_ESM.docx]

1. Search terms for MEDLINE^®^

Table 3.Search terms for MEDLINE^®^

| **Search No.** | **Search Terms** |
| --- | --- |
| **1** | **Title or abstract:** Ornithine transcarbamylase deficiency OR OTC deficiency OR Ornithine carbamoyltransferase deficiency disease |
| **2** | **Title or abstract:** sign OR symptom OR impact OR burden OR quality of life OR QOL OR HRQOL |
| **3** | **Title or abstract:** scale OR measure OR rating OR tool OR instrument OR questionnaire OR index OR assessment OR diary OR outcome OR PRO OR patient report OR COA OR clinical outcome assessment OR qualitative OR interview OR focus group |
| **4** | **1 AND 2** |
| **5** | **1 AND 3** |
| **Filters:** Published between 2009-2019; English language; Human | |

1. Concept elicitation interview prompts

| **Signs and symptoms of OTCD** |
| --- |
| - Are you familiar with the term Ornithine Transcarbamylase (OTC) deficiency? - Are you familiar with the term **hyperammonemia**?   - If yes, what does hyperammonemia mean to you?      - Do you know when [you/your child] [are/is] experiencing it? - Have/has [you/your child] ever experienced a **hyperammonemia event** due to [your/your child’s] *[participant’s term for OTCD]*?   - How many **hyperammonemia events** have [you/your child] had?   - Can you describe what that event(s) was like? - In addition to the symptoms you already told me about *[list signs and symptoms]*, do [you/your child] experience any other symptoms of *[participant’s term for OTCD]*?  \| **Note:** For each sign/symptom the participant describes above, ask the following questions if it has not already been spontaneously reported by the participant: \| \| --- \|  \| - *[For patients]:* Can you tell me more about how *[symptom]* **feels?** *[For caregivers]:* How does your child describe *[symptom]* **feeling**?   - How would [you/your child] describe *[symptom]* to someone who has never experienced it?   - *[For caregivers]:* How do you know your child is experiencing *[symptom]*? Do you observe it or do they tell/report it to you? - How do you know [your/your child’s] *[symptom]* is **related** to *[participant’s term for OTCD]*? - Does the *[symptom]* occur at a certain time of day? Under certain circumstances? Is there a pattern?   - Does *[symptom]* occur before **hyperammonemia events**?   - Does *[symptom]* occur during **hyperammonemia events**?   - Does *[symptom]* occur after **hyperammonemia events**? - **How often** does *[symptom]* occur?   - Within a day? Within a week? Within a month? - **How long** does *[symptom]* last? \| \| --- \|  \| **Note:** If any of the following symptoms has not already been spontaneously reported by the participant, probe the following. Unless otherwise noted, ask all participants (including caregivers) all probes. \| \| --- \|  - Do/does [you/your child] experience any of the following symptoms due to [your/your child’s] *[participant’s term for OTCD]*?  \| **PROBE:** \| \| \| --- \| --- \| \|  \| Confusion \| \|  \| Difficulty focusing or paying attention \| \|  \| Poor balance \| \|  \| Stomach pain \| \|  \| Headache \| \|  \| Tiredness \| \|  \| *[For adults and adolescents:]* Loss of appetite \| \|  \| - *[For children:]* Not feeling hungry when it is time to eat \| \|  \| *[For adults and adolescents:]* Nausea \| \|  \| - *[For children:]* Feeling like [you/your child] are/is going to throw up \| \|  \| *[For adults and adolescents:]* Vomiting \| \|  \| - *[For children:]* Throwing up \| \|  \| Dizziness \| \|  \| *[For adults and adolescents:]* A sudden change in how [you/your child] felt (for example, feeling drunk or disoriented) \| \|  \| - *[For children:]* A sudden change in how [you/your child] felt (for example, feeling different than [you/your child] normally do/does) \| |
| **Most important to treat signs and symptoms** |
| *[For adults, caregivers and adolescents:]*   - Of all the symptoms that [you/your child] experience *[list symptoms]*, which are the most important to treat? Why?   *[For children:]*   - Of all the symptoms that you experience *[list symptoms]*, which ones would you want a medicine to treat the most? Why? |
| **OTCD Impacts** |
| - *[If participant previously mentioned impacts:]* Earlier you mentioned *[impact]*, could you please tell me a little more about that? - How (else) does *[participant’s term for OTC]* affect [your/your child’s] life?  \| **Note:** If any of the following impacts has not already been spontaneously reported by the participant, probe the following: \| \| --- \|  - Does *[participant’s term for OTC]* cause [you/your child] to:   - Feel limited by what [you/your child] can eat   - Feel scared [you/your child] will have a hyperammonemia event   - Feel different from others   - Feel embarrassed   - Feel bad about [yourself or himself/herself]   - Have difficulty performing physical activity   - Have difficulty falling asleep |
| **Most important to improve impacts** |
| *[For adults, caregivers and adolescents:]*   - Of all the impacts that [you/your child] experience *[list impacts]*, which are the most important to treat? Why?   *[For children:]*   - Of all the ways your OTC deficiency impacts you *[list impacts]*, which ones would you want a medicine to treat the most? Why? |
